# Vitamin D levels in peanut allergic children

**DOI:** 10.1186/1710-1492-7-S2-A8

**Published:** 2011-11-14

**Authors:** Adam Fowlie, Laura Kim, Trefford L Simpson, Harold Kim

**Affiliations:** 1 University of Western Ontario, Canada; 2 University of Waterloo, Canada; 3 McMaster University, Canada

## Background

The prevalence of peanut allergy is increasing. The reasons for this are not entirely known. A factor may be vitamin D (Vit D).

## Methods

This study was performed in a referral allergist’s office in Ontario. Prospectively, all patients (<18 years old) with peanut allergy who were tested for peanut specific IgE (PN IgE) also had Vit D measured. All measurements were done between December 2010 and May 2011. The Vit D measure was 25-hydroxy vitamin D. Patients were divided into three groups: deficient (less than 25 nmol/L), insufficient (25-75 nmol/L) and sufficient (75-250 nmol/L). Vit D levels were compared to PN IgE, sex, age, body mass index (BMI) and other allergies.

## Results

Fifty peanut allergic patients were included. The mean Vit D level of the patients was 73.8 nmol/L and the 95% confidence interval was 69.6 - 75.7 nmol/L. One patient (2%) had deficient and thirty-one (62%) of the patients had insufficient Vit D levels. Nineteen (38%) had Vit D levels in the sufficient range. There was no correlation between Vit D levels and PN IgE or BMI. Generalized linear modeling showed that vit D levels were predicted by age and sex (p=0.04 & p=0.002, respectively).

## Conclusions

Two percent of our patients had deficient Vit D levels while 62% of our patients had insufficient Vit D levels. These levels were statistically associated with age and sex. Insufficiency of Vit D may play a role in peanut allergy.

